# Effects of plyometric jump training on measures of physical fitness and lower-limb asymmetries in male soccer players according to maturity: A randomized controlled trial

**DOI:** 10.1371/journal.pone.0340863

**Published:** 2026-01-15

**Authors:** Nizar Bouafif, Roland van den Tillaar, Abdelkader Mahmoudi, Anis Chaouachi, Raouf Hammami

**Affiliations:** 1 University Of Manouba, Higher Institute of Sport and Physical Education of Ksar-Said, University Campus, Manouba, Tunisia; 2 Tunisian Research Laboratory ‘Sports Performance Optimization’, National Center of Medicine and Science in Sports (CNMSS), Tunis, Tunisia (CNMSS-LR09SEP01); 3 Department of Sport Sciences and Physical Education, Nord University, Levanger, Norway; eCampus University: Universita degli Studi eCampus, ITALY

## Abstract

The purpose of this study was to determine the effects plyometrics on dynamic balance, muscle power and lower limb asymmetries in youth soccer players according to maturity. 43 youth soccer players (23 pre pubertal and 20 post pubertal) were classified according to their peak height velocity (PHV) and participated in a 8-week progressive plyometrics training program or an active control with two weekly sessions, alongside three regular soccer-specific training sessions per week. Participants’ training and performance caliber was rated Tier 2. While plyometrics training groups performed jumping and hopping exercises such as maximal and submaximal hopping on stable ground, active control groups performing passing skills. Pre and post training, tests included the Y balance test, single leg hop and 505 change-of-direction speed (CoD) tests; and lower limb asymmetries were calculated based upon these tests. The main findings were that plyometrics significantly improved these physical attributes, with greater enhancements observed in the post-Peak Height Velocity (PHV) group compared to both the pre-PHV group and the active control group. However, asymmetry did not improve after the training intervention and even increased for balance test in the post-PHV training groups compared with the pre-PHV groups at the post test. Plyometrics effectively enhances jumps, CoD and dynamic balance, with greater benefits observed in post- PHV male soccer players. While overall lower-limb asymmetry largely remained unchanged, a notable finding was an increase in balance asymmetry specifically within the post-PHV group, attributed to a differential enhancement of left and right limb balance.

## Introduction

Lower-limb asymmetries are common in youth soccer players due to the sport’s inherently unilateral demands, such as frequent kicking, cutting, and pivoting [1,2]. These asymmetries, often manifested as imbalances in strength, power, or neuromuscular control between limbs, have been linked to impaired performance and elevated injury risk, particularly during dynamic actions such as jumping, change of direction (CoD), and balance-related tasks [3,4]. Therefore, early identification and targeted interventions to mitigate such asymmetries are critical components of long-term athlete development programs. However, there remains a lack of research examining asymmetry across different stages of maturation using ecologically valid and practically applicable screening tools.

Plyometric jump training, which consists of dynamic and explosive exercises such as hops, bounds, and jumps, has gained attention as an effective method to improve neuromuscular control, inter-limb coordination, power output, and balance [5,6]. Recent studies suggest that both bilateral and unilateral plyometric protocols can reduce strength and power asymmetries by targeting deficits in both the dominant and non-dominant limbs [7,8]. Improvements in inter-limb symmetry are thought to occur through enhanced motor unit recruitment, synchronization, and proprioceptive feedback, which in turn benefit balance and CoD abilities-key physical attributes in soccer [9,10].

For instance, Sammoud et al. [5] reported significant reductions in inter-limb asymmetry following an 8-week plyometric program in Tier 2 prepubertal male soccer players, defined as trained or development-level athletes with regular structured practice and competition experience but not yet at elite or professional levels [11]. This classification follows the framework proposed by McKay et al. [11], which categorizes athletes based on training history, competitive standard, and performance level to ensure consistency across sport science research. However, such findings have not been widely replicated across different maturational stages. Given that training adaptations are influenced by maturity status, it is important to determine whether the efficacy of plyometric training in reducing asymmetry and enhancing physical fitness is consistent across pre-, circa-, and post-peak height velocity (PHV) groups.

Youth athletes undergo rapid neuromuscular, hormonal, and morphological changes during growth, which can affect their responsiveness to training [12,13]. Prepubertal athletes (pre-PHV), for instance, tend to experience more pronounced neural adaptations and improvements in CoD and balance following plyometric interventions, largely due to heightened neural plasticity and lower baseline training status [7,14]. In contrast, post-PHV players may respond better in terms of muscle hypertrophy and maximal power output [15,16]. A recent meta-analysis by Ramirez-Campillo et al. [9] confirmed that youth athletes, regardless of maturity, benefit similarly from plyometric training in most physical fitness outcomes except for CoD, which improves more significantly in prepubertal players. These findings underscore the need for maturity-specific programming to optimize adaptations.

Furthermore, the estimation of maturity status through PHV is central to such investigations. PHV represents the period of maximum growth rate in stature during adolescence and serves as a noninvasive indicator of biological maturity. Among various approaches, the Mirwald et al. (2002) maturity offset equation remains the most commonly applied method in youth sport research due to its practicality and strong validation across diverse populations [17]. Other techniques—such as skeletal age assessment (e.g., Tanner-Whitehouse method) and secondary sexual characteristic evaluation (Tanner staging) provide more direct measures but are invasive or impractical in field settings [18]. While alternative Mirwald et al. (2002) maturity offset equation have been proposed to refine accuracy in specific cohorts, the Moore et al. (2015) method continues to be preferred for estimating years from PHV in large-scale or field-based studies of youth athletes because it balances feasibility and reliability [Moore et al. (2015]. Clarifying the chosen approach to estimate PHV enhances the interpretability and comparability of findings across studies examining maturation-related differences in training responses.

Furthermore, recent studies emphasize the importance of using field-based functional tests, such as the single-leg countermovement jump, Y-balance, and 505 CoD test, to assess asymmetries and monitor performance in a practical, reliable, and sport-specific manner [11,19,20]. These tests are not only predictive of asymmetry-related injury risk but are also sensitive to detecting changes in functional performance across different maturity levels. Notably, Read et al. [10] suggested that asymmetries may emerge early in development and remain relatively stable, advocating for early, ongoing interventions beginning in the pre-PHV stage to prevent long-term performance limitations and injury risk [17,18].

Collectively, these findings highlight the importance of tailoring plyometric programs to biological maturity and incorporating asymmetry monitoring as part of comprehensive youth soccer training.

Therefore, the present study aimed to investigate the effects of an 8-week plyometric jump training program versus active control on measures of physical fitness (dynamic balance, unilateral horizontal jumping, and CoD speed) and lower-limb asymmetries in Tier 2 youth soccer players, stratified by maturity status. Based on existing literature [9,12,13], we hypothesized that prepubertal players would exhibit greater improvements in balance and CoD, as well as more pronounced reductions in lower-limb asymmetry [10] compared to their more mature counterparts.

## Materials and methods

### Subjects

Participants were recruited from local soccer club of Takelsa, Tunisia. Recruitment was conducted over a period of two and a half months, from 15/03/2025 to 30/05/2025.The sample size estimation was computed using G*Power software (version 3.1.6). Based on findings from Sammoud, Negra (5) examining the effects of plyometric jump training on lower limb asymmetry predicted from the single leg hop test in youth soccer players (Cohen’s f = 0.42), an a priori power analysis with a type I error of 0.05 and 80% statistical power was computed. The analysis indicated that a total of 10 participants per group would represent a sufficient sample. Accordingly, 43 youth soccer players (23 pre pubertal and 20 post pubertal) were recruited from a youth soccer Center of Takelsa, Nabeul, Tunisia, to participate in this study (Table 1). Participants’ biological maturity status was estimated using the maturity offset method based on the prediction equation of Moore et al. [17]. This equation was selected because it provides an updated and more accurate estimation of years from peak height velocity (PHV) compared with the earlier Mirwald equation, particularly in youth populations where the original model tended to overestimate maturity in younger athletes and underestimate it in older ones. The Moore equation has been validated in both male and female adolescent athletes across various sports, making it suitable for the present sample. Nevertheless, as with all non-invasive predictive approaches, it provides an estimate rather than a direct measure of biological maturity and may still be subject to individual variation. According to McKay, Stellingwerff (20) definition of training and performance caliber, the study population can be categorized Tier 2 (trained/developmental). Participants were recruited from two regional soccer academies competing at the Tier 2 level of national youth competition. To be eligible for inclusion, players had to meet the following criteria: a minimum of 2 years of continuous, structured soccer training in an academy or club environment, with at least three training sessions per week, no participation in systematic strength or plyometric training in the 6 months prior to the intervention, no musculoskeletal injuries, surgeries, or neurological conditions in the past 6 months that could interfere with performance. Full participation in all training and testing sessions, with no more than 10% absence allowed during the 8-week intervention period; and no concurrent involvement in other sports at a competitive level.

**Table 1:** Anthropometrics of the examined study cohort according to group allocation.

	Pre-PHV		Post-PHV	
Training group	Plyometrics (n = 10)	Control (n = 12)	Plyometrics (n = 10)	Control (n = 10)
Age (yrs.)	8.7 ± 0.6	8.8 ± 0.8	13.4 ± 0.3	13.4 ± 0.3
Height (m)	1.21 ± 0.10	1.22 ± 0.09	1.56 ± 0.08	1.51 ± 0.07
Body mass (kg)	25.3 ± 4.9	26.6 ± 4.8	39.8 ± 7.6	38.3 ± 4.2
PHV	−2.5 ± 0.4	−2.4 ± 0.3	1.3 ± 0.3	1.5 ± 0.2

Players were stratified into pre-PHV and post-PHV groups based on maturity offset values calculated using the predictive equation of Moore and McKay [17]. A cut-off of ±0.5 years from the estimated age at PHV was used to distinguish between groups, with players showing a maturity offset ≤ −0.5 years classified as pre-PHV and those ≥ +0.5 years classified as post-PHV. Following stratification, participants within each maturity group were randomly assigned to either the plyometric training or control condition using a stratified randomization procedure (computer-generated random numbers), ensuring balanced group sizes by maturity status. Allocation was performed by an independent researcher not involved in data collection or training supervision to maintain allocation concealment.

Pre-study screening questionnaires completed by players and coaches indicated that most pre-PHV participants had approximately 2–3 years of organized soccer training experience, whereas post-PHV players typically had 4–5 years. None of the participants had previously engaged in structured resistance or plyometric training, ensuring a relatively homogeneous neuromuscular training background across groups.

All participants were active members of their respective teams and followed similar weekly training routines that included technical-tactical drills, small-sided games, and match play under the supervision of licensed coaches. Before study participation, the young players and their parents or legal representatives received a letter containing information about potential benefits and risks of study participation. Written parents or legal representatives and athletes signed the consent form after thorough explanation of the objectives and the scope of the study, the procedures, risks, and benefits. The study was conducted according to the latest version of the Declaration of Helsinki and the protocol was fully approved by the Local Clinical Research Ethics Committee (Personal Protection Committee) under the following code (N°: 0221//2024) before the commencement of any assessments. None of the participating athletes had a history of psychological and musculoskeletal, neurological, or orthopedic disorders six months prior to the start of the study.

### Procedure

**Fig 1:**
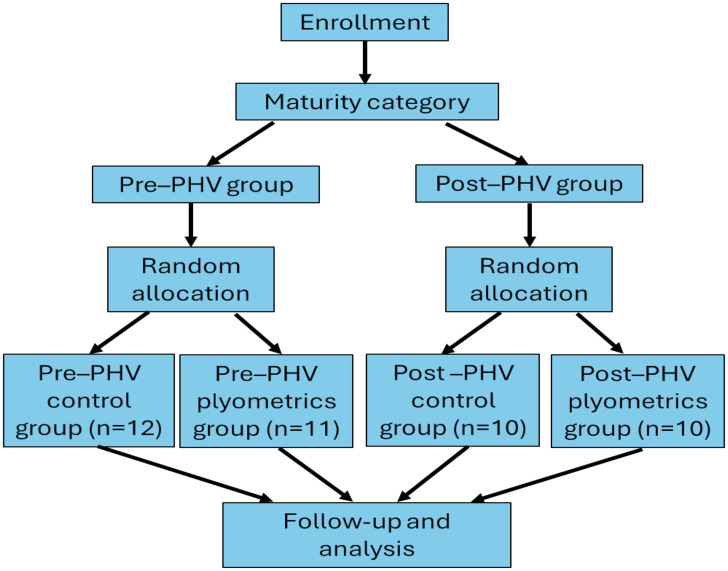
Flow chart.

All performance tests were conducted both before (pre-test) and after (post-test) the 8-week training intervention for all participants and across all outcome measures**.** Pre-testing was carried out during the week prior to the start of the intervention, while post-testing was conducted during the week immediately following the final training session, using the same procedures and testing order. One week before the start of the study, a familiarization session was scheduled to allow the participating athletes to become acquainted with the applied tests and exercises. Participants of the plyometrics groups received instructions on the proper exercise techniques. Pre and post training, athletes performed dynamic balance (Y-Balance), single leg hop jump and a 4 × 5 meters change-of-direction speed tests. Before testing, a standardized warm-up was performed consisting of submaximal running for 5 min, submaximal strength exercises (e.g., 10 bench press, 3–5 upper countermovement jumps [CMJ]) and dynamic stretching exercises (Fig 1). All tests were separated by a 5–10 minutes rest period. Each test was performed two times and rest between test trials was three minutes. The best trial was used for further statistical analyses. The same test sequence was applied during pre and post-tests.

### Measurements

Athletes’ body height and mass were collected using a wall-mounted stadiometer (Florham Park, NJ) and an electronic scale (Baty International, West Sussex, England), respectively. The sum of skinfolds was assessed using Harpenden’s skinfold calipers. Anthropometric testing was conducted according to Deurenberg, Weststrate [21] who reported similar prediction errors between adults and adolescents. Thereafter, biological maturity was evaluated non-invasively using chronological age, standing and sitting height as input parameters for a regression equation to subsequently predict the maturity offset [17]. The equation has previously been validated for boys and presents a standard error of estimate reported as 0.542 years [17].

Dynamic balance. Dynamic balance was assessed using the Y-balance test (YBT) according to a previously described protocol [22], which has been shown to be reliable for youth soccer players (ICC = 0.92) [14]. Testing was conducted barefoot. Participants stood on the dominant leg with the most distal aspect of their big toe on the center of the footplate from the YBT kit. The participants were then asked to push the reach-indicator block with the free limb in the anterior, posterior medial, and posterior lateral directions while maintaining their single-limb stance on the central footplate [23]. Participants were not allowed to lift the heel of the stance leg during the test performance. Maximal reach distances were recorded to the nearest 0.5 cm of the Y-Balance Test Kit™ (Functional Movement Systems, Inc., Danville, VA, USA). The trial was repeated if participants moved the stance leg or failed to return the reaching foot to the starting position. A composite score was calculated and considered as the dependent variable using the following formula: composite score-YBT (%) = ([maximum anterior reach distance + maximum posteromedial reach distance + maximum posterolateral reach distance]/[leg length x 3]) × 100.

The single leg hop test for distance for the right leg and left leg was conducted, while participants adopted a standing position on the designated testing leg, with their hands on hips and their toes behind the starting line. Subjects were then instructed to hop forward as far as possible and land on the same leg. Upon landing, participants were required to ‘hold and stick’ their position for ~2 s. The leg used to kick a soccer ball was identified as the dominant leg [14]. The distance was tested to the nearest centimeter using a tape measure. The best out of two valid trials was recorded for further analysis. The between-trials ICCs were 0.94 and 0.91 for right and left legged jumps, respectively.

In the 505 change of direction (CoD) test with turning to left or right, the players positioned themselves in a standing stance, starting from a point 10 m away from the designated start line. Upon the start signal, they sprinted as quickly as possible through the start/finish line. At the 15-m mark, indicated by a cone marker, the players executed a 180° pivot and immediately returned to the start line, aiming to complete the test in the shortest time possible [24]. To ensure proper execution of the test, a researcher was positioned at the turning line. If a participant changed direction before reaching the designated turning point or turned with the incorrect foot, the trial was disregarded, and the participant was allowed to reattempt the trial after a recovery period. The performance time was automatically recorded using photocell gates (Brower Timing Systems, Salt Lake City, UT, USA; accuracy of 0.01 s) positioned 0.4 meters above the ground. A rest period of three minutes was provided between each trial to minimize fatigue. Two trials with each turning foot were conducted, and the best performance achieved among the two attempts was selected for further analysis. The reliability of the measurements between trials was assessed using the ICC, which yielded a value of 0.90, indicating a high level of consistency between the trials.

Bilateral asymmetries were calculated from the performance measure during the Y-balance, single leg hop and 505 CoD tests. A negative sign (−) was arbitrarily assigned when the left leg was the stronger one, and a positive sign (+) was used when the right leg was the stronger one. In the literature [25], relative inter-limb asymmetry for the lower limbs was determined the formula: (Right leg − Left leg)/(Right leg + Left leg) 100. Test–retest reliability scores for these measures from the present results have been shown good with our study (ICC = 0.73).

### Training program

The plyometric programs were designed in accordance with the long-term athletic development prescription strategies proposed by Granacher et al. [7] and were implemented during the 2023 in-season period (February–March). Both pre- and post-PHV groups completed an 8-week in-season program consisting of two sessions per week, in addition to their regular soccer training, which primarily focused on developing sport-specific technical and tactical skills. Plyometric training was integrated within the players’ regular 90-minute soccer sessions and conducted twice weekly, constituting a form of concurrent training in which neuromuscular (plyometric) and technical–tactical (soccer-specific) stimuli are combined within the same training cycle. This structure reflects the ecological conditions of youth soccer and aligns with evidence showing that concurrent training can promote complementary gains in neuromuscular power and endurance when appropriately sequenced and monitored [26,27].

Each session began with a standardized 10-minute dynamic warm-up that included submaximal running, dynamic stretching, multidirectional low-intensity movements (forward, sideways, backward), acceleration runs, and preparatory jumps. Following the warm-up, athletes in the intervention groups performed plyometric exercises characterized by fast and explosive stretch–shortening cycle (SSC) actions, minimal ground contact times, and progressive overload, as described by Davies et al. [28]. The control groups performed a matched-duration passing drill.

The plyometric protocol, adapted from Bogdanis et al. [29] and in line with youth training guidelines [26,27], included six lower-limb exercises per session targeting both vertical and horizontal force production (bilateral and unilateral 20-cm drop jumps, standing long jumps, and lateral bounds). Exercises were performed bilaterally and unilaterally, as appropriate, with ground contact times kept below 250 ms for all fast SSC drills, verified by coach observation and auditory feedback. Proper landing control and jump height were emphasized to ensure effective SSC utilization and safe mechanics.

Training intensity and progression followed a progressive overload model, increasing the number of foot contacts (from 54 to 120 per session across the 8 weeks), jump height (from 20 to 30 cm for drop jumps), and task complexity (from bilateral to unilateral and multidirectional movements). Athletes performed 3–4 sets of 5–10 repetitions per exercise, with 30–60 seconds of rest between sets (Table 2). Each repetition was executed at maximal voluntary effort, emphasizing explosive take-off and minimal ground contact time.

**Table 2:** Progression over 8-weeks of plyometrics.

Week	1	2	3	4	5	6	7	8
**Bilateral jumps**								
Drop jump	3 × 6	3 × 8	4 × 8	4 × 10				
Horizontal jumps	3 × 6	3 × 8	4 × 8	4 × 10				
Lateral hops	3 × 6	3 × 8	4 × 8	4 × 10				
Unilateral 20-cm drop jump					3 × 3	3 × 4	4 × 4	4 × 5
**Unilateral jumps (each leg)**								
Unilateral horizontal jumps					3 × 3	3 × 4	4 × 4	4 × 5
Unilateral lateral hops					3 × 3	3 × 4	4 × 4	4 × 5
Total foot contact	54	72	96	120	54	72	96	120

To address potential differences in total training load between groups, both internal and external loads were carefully monitored. The session rating of perceived exertion (sRPE) method (Foster et al., 2001) was used after each session to quantify internal load. The duration and structure of the plyometric and control sessions were matched (~25 minutes). External load in the experimental groups was further tracked by recording the total number of jump contacts per session and progression across weeks. No significant between-group differences in total session RPE were observed, indicating comparable perceived effort across training modalities.

After completing the plyometric or passing component, all players continued with 60 minutes of identical soccer-specific training (30 minutes of technical–tactical drills and 30 minutes of small-sided games), followed by a 5-minute cooldown. The control groups performed passing drills (e.g., two-touch triangle and square patterns, low-driven passes) for the same duration as the plyometric component (Table 3). These drills were designed to maintain moderate cardiovascular demand (heart rates: 120–140 bpm), ensuring similar overall physiological load to the plyometric sessions. Consequently, total training exposure, duration, and internal load were matched across groups, minimizing potential confounding effects related to unequal workload.

**Table 3:** Exemplified of training programs.

Training group	Plyometrics	Control
**Warm-up**	5 min Activation: submaximal running, low intensity forward, sideways, and backward running, several acceleration runs, jumping and dynamic stretching.	
**Intervention**	Plyometrics; (2 sessions/week) 1-8 weeks of 3–4sets of 5–10 reps	Soccer training; (2 sessions/week) 8 weeks of soccer-specific ball passing skills
**Soccer specific training**	60 min technical and tactical skills	
**Cool down**	5 min	
**Total training volume**	**90 min**	

All sessions were supervised by two qualified strength and conditioning specialists who provided real-time feedback on technique, posture, landing mechanics, and intensity to ensure safe execution and adherence to correct plyometric principles throughout the intervention.

### Statistical analysis

Data are presented as means and standard deviations (SD). Normality assumption was tested using the Shapiro-Wilk test. To establish the effects of the two training programs on the dependent variables, a 2 (age: pre-post PHV) x 2 (training: plyometrics-control) x 2 (time: pre post training repeated measures) ANOVA with repeated measures was determined for the performances and asymmetry in jumps, CoD and balance. In case group-by-time interactions reached the significance level (p < 0.05), group-specific post-hoc tests (i.e., Holm-Bonferroni adjusted paired t-tests) were calculated. Effect size was evaluated with η^2^ (ETA squared), where 0.01 < η^2^ < 0.06 constitutes a small effect, 0.06 < η^2^ < 0.14 constitutes a medium effect, and η^2^ > 0.14 constitutes a large effect [28]. At baseline, two trials were used for each variable to analyze intra-session reliability [30]. Test-retest reliability was assessed using data from all participants. ICCs were interpreted as follows: < 0.40 = poor, 0.40–0.70 = fair, 0.70–0.90 = good, and >0.90 = excellent [31]. The alpha level of significance was set at p < 0.05. All analyses were conducted in JASP (version 0.18.1, Amsterdam, Netherlands).

## Results

**Fig 2:**
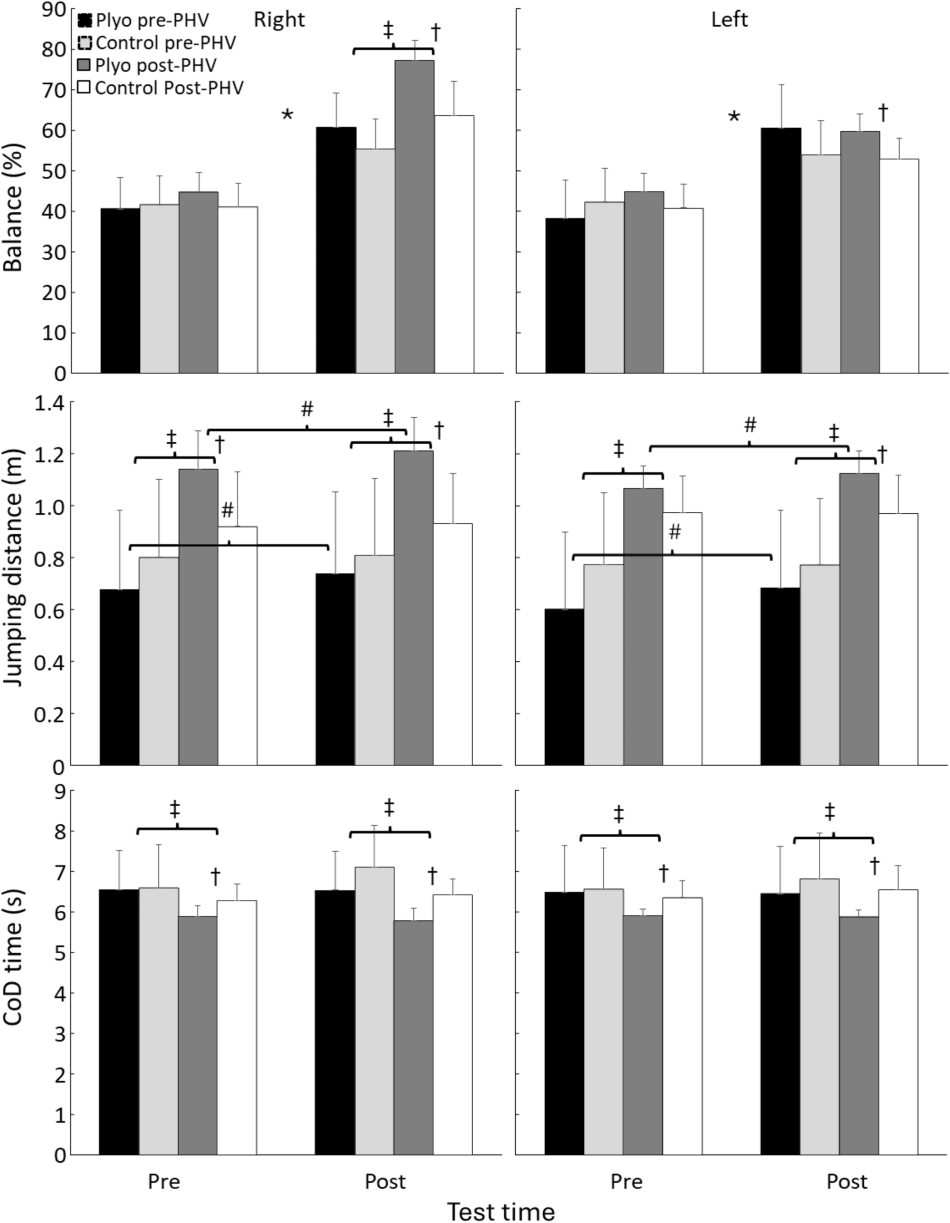
Balance, jump and CoD performance of the different groups and pre and posttest. * indicates a significant change from pre to posttest for all groups. # indicates a significant change from pre to posttest for this group. † indicates a significant difference between the plyometrics and control group in this test. ‡ indicates a significant difference between the pre- and post-PHV group in this test.

All performances were significantly enhanced from pre- to post test (F ≥ 4.3, p ≤ 0.045, η2 ≥ 0.10) except not for the CoD with right foot (F = 0.21, p = 0.65, η2 < 0.01). Also, a significant time × training group interaction effect was found for all tests (F ≥ 7.2, p ≤ 0.010, η2 ≥ 0.16) together with significant age group effect for all jump tests and for the balance and CoD test with the right foot (F ≥ 4.2, p ≤ 0.048, η2 ≥ 0.10). Only balance with the right foot showed a significant training group and time × age interaction effect (F ≥ 8.1, p ≤ 0.007, η2 ≥ 0.17), while only a significant training group × age group interaction effect for jumps with right foot and a time × training group × age group interaction effect for the CoD with right foot. Post hoc comparison revealed that balance left and right increased for all groups from pre- to post, but for the right foot the post-PHV group at the post-test reached better balance results than the pre-PHV groups. This was especially the result of better results in the plyometrics post-PHV groups, which have significantly better balance in both feet than the control post-PHV group at the posttest (Fig 2).

Jump performance was always better for the post-PHV group compared to the pre-PHV group. Furthermore, did both plyometric groups increased jump performance significantly over time, resulting in longer jumps at the posttest compared to the control groups (Fig 2). Also, in the CoD times the times (left and right foot) were always better for the post -PHV group compared with the pre-PHV group, but at the post-test the post-PHV plyometrics group had ran significantly faster than their equally aged control group (Fig 2).

About asymmetry, balance asymmetry was effected by training age (F = 15.0, p < 0.001, η2 = 0.29), test time (F = 35.5, p < 0.001, η2 = 0.45) together with test time × training age interaction (F = 40.9, p < 0.001, η2 = 0.51) and test time × training age × training group interaction (F = 7.2, p = 0.011, η2 = 0.16), while the rest was not significant (F ≤ 2.5, p ≥ 0.119, η2 ≤ 0.06). Jumping asymmetry was only significantly affected by training group (F = 6.5, p = 0.015, η2 = 0.14), while training age almost reached significance level (F = 3.96, p = 0.054, η2 = 0.09), and no other effects reached significance level (F ≤ 1.85, p ≥ 0.18, η2 ≤ 0.045). No significant effects were found for change of direction over time, training and age (F ≤ 3.1, p ≥ 0.086, η2 ≤ 0.07).

**Fig 3:**
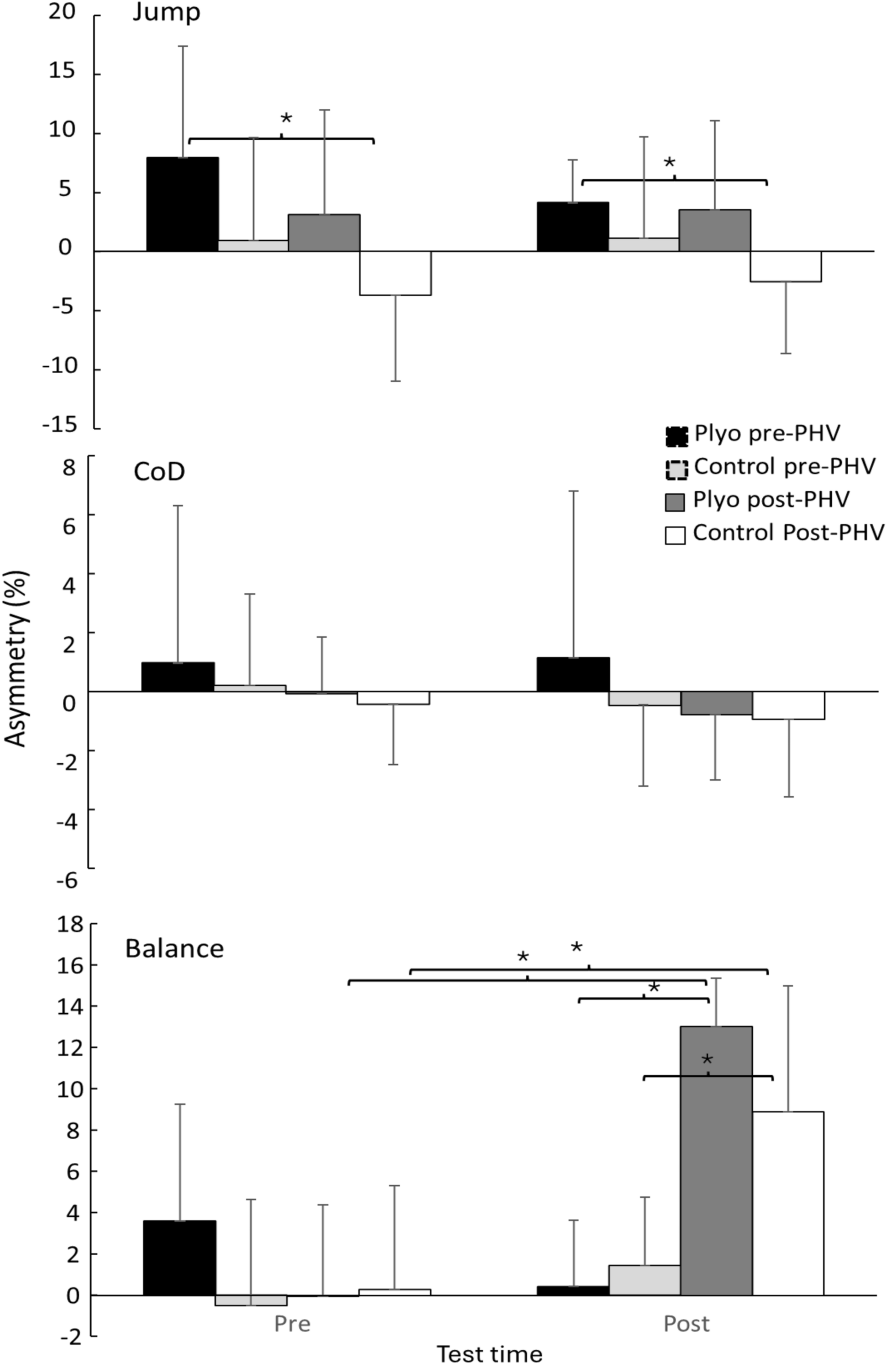
Asymmetry between left and right foot performance of jumps, balance and change of direction before and after a training period for plyometric and control groups in pre- and post- PHV youth soccer players. * indicates a significant difference between these two groups.

Post hoc comparison revealed that for balance post hoc comparison revealed that asymmetry increased significantly in both training groups of the post -PHV youth soccer players from pre to post testing, while no significant changes were observed in the pre-PHV group, resulting in significantly higher asymmetry in posttest between the post-PHV groups with the pre-PHV groups (Fig 3). Asymmetry in jump performance was significantly different between the plyometrics training group of the pre-PHV group at pre and post with the control group of the post-PHV group (Fig 3).

## Discussion

This study examined the effects of an 8-week plyometric training program on dynamic balance, single-leg hop performance, change of direction (CoD) speed, and lower-limb asymmetries in Tier 2 male soccer players across different maturity statuses. The results revealed that plyometric training significantly improved all physical performance measures, with greater enhancements observed in the post-peak height velocity (post-PHV) group compared to both the pre-PHV group and the active control group. However, contrary to our expectations, asymmetry in dynamic balance did not improve and even increased in the post-PHV groups, particularly for balance tasks. These findings underscore the importance of considering biological maturation when designing and prescribing plyometric interventions [12,32,33].

The observed improvements in jump and CoD performances align with findings from prior systematic reviews and meta-analyses [9,34,35], which consistently report moderate to large effect sizes for these outcomes following plyometric interventions in youth. The superior responsiveness observed in post-PHV players is consistent with maturation-related neuromuscular adaptations, such as enhanced motor unit recruitment, greater neural conduction velocity, and increased muscle stiffness and reactive strength—all of which are critical to explosive actions like jumping and rapid directional changes [5,15]. These physiological advantages, combined with higher mechanical tolerance and hormonal influences during adolescence, enable more mature athletes to benefit more from high-intensity training stimuli [7,13].

In contrast, findings related to dynamic balance were more complex. While balance improved in both age groups post-intervention, a larger improvement was observed in the post-PHV group’s dominant leg, potentially contributing to an increase in inter-limb asymmetry. This differs from the conclusions of meta-analyses by Ramachandran and Singh [36] and Clemente and Ramirez-Campillo [37], which suggested either modest or no significant balance gains from plyometric training across age groups and sexes. The greater gains in dominant-leg balance in the post-PHV athletes may stem from developmental factors—such as rapid changes in body morphology during PHV, which challenge postural control and force the neuromuscular system to recalibrate [38,39]. As athletes mature, experience, motor learning, and neuromuscular control of the dominant leg may become more refined, facilitating superior adaptation to training stimuli [34].

This maturity-dependent improvement, however, might also explain the unexpected increase in asymmetry in balance post-intervention. As plyometrics enhances force-generating capacities and neuromuscular activation, these improvements may occur unequally between limbs, especially in unilateral or dominant-leg-biased movements common in soccer and some plyometric exercises [40]. In more mature athletes, the enhanced ability to generate force and react quickly could lead to disproportionate neuromuscular adaptations between limbs, revealing or even exacerbating subtle pre-existing asymmetries [5,10].

Furthermore, the nature of the balance assessments may have contributed to these findings. Unlike jump or CoD tests that primarily rely on maximal force or speed output, dynamic balance tasks demand complex integration of sensory feedback, motor planning, and neuromuscular coordination [39,41]. If plyometric training disproportionately enhanced strength or power without concurrently improving inter-limb coordination or proprioception, it might have temporarily disrupted equilibrium control, especially in athletes who gained more strength in their dominant leg. Pre-PHV athletes, with a less differentiated neuromuscular system, may have responded with more global, balanced adaptations due to a lower training threshold and less limb dominance [7,42].

Although the plyometric training program was designed with age-appropriate intensity and strict supervision, the inclusion of young pre-PHV players (mean age 8.7 years) warrants careful consideration regarding developmental readiness and safety. At this stage, the musculoskeletal and neuromuscular systems are still developing, and excessive or poorly supervised training may increase the risk of overuse injuries or fatigue-related compensations [Lloyd & Oliver, 2012; Faigenbaum et al., 2016]. Therefore, early plyometric or resistance training for prepubertal children should prioritize movement competency and quality of execution over intensity or volume. Consistent with current youth strength and conditioning frameworks [Granacher et al., 2018; Behm et al., 2017], the present program emphasized low-to-moderate intensity drills (e.g., drop jumps ≤30 cm, bilateral first, then unilateral), progressive overload through gradual increases in contacts and complexity rather than external load, and strict technical supervision during all exercises. Recovery between sessions and between sets (≥48 hours, ≥ 30–60 s rest) was ensured to prevent excessive fatigue. The integration of plyometric exercises within normal soccer practice further reduced the likelihood of overload, ensuring ecological relevance and safety. Nevertheless, while the total weekly training frequency matched the academy’s schedule, it may still be relatively high for 8–9-year-olds, highlighting the importance of individualized monitoring using simple tools such as rate of perceived exertion (RPE), jump counts, and observation of technique quality to ensure appropriate recovery and progression. Future studies should quantify internal and external training loads to better clarify the relationship between stimulus, recovery, and adaptation in prepubertal athletes.

From a practical perspective, this study provides valuable guidance for coaches and practitioners working with youth soccer players. Plyometric training appears effective and safe for both pre- and post-PHV athletes when appropriately scaled, but its effects differ according to maturity status. Pre-PHV players may benefit primarily through improvements in coordination and balance, whereas post-PHV players demonstrate greater enhancements in jump and CoD performance. However, in more mature players, close attention should be paid to potential asymmetry, particularly in balance performance, as gains may be uneven across limbs. Coaches should integrate bilateral and proprioceptive training elements and periodically monitor asymmetries using simple, reliable tools (e.g., Y-Balance Test, single-leg hop) to guide individualized programming and reduce injury risk.

It is important to interpret the observed reductions in inter-limb asymmetry among pre-PHV players with caution. In children around 8 years of age, lower-limb asymmetries are common and often reflect normal neuromuscular variability associated with rapid growth, uneven segmental development, and transient coordination deficits rather than maladaptive imbalances [1–3]. Therefore, while the improvements observed in the current study may suggest enhanced inter-limb coordination and neuromuscular control, part of these changes could also be attributed to natural maturation processes rather than the plyometric intervention alone. Furthermore, a detailed examination of asymmetry direction revealed that, in most cases, improvements were primarily driven by performance gains in the non-dominant limb, consistent with previous evidence indicating that the weaker limb tends to exhibit greater adaptive potential due to its lower baseline strength and coordination [4–6]. These findings underscore the importance of accounting for maturational variability when interpreting training-induced changes in asymmetry among prepubertal athletes.

This study has several limitations. First, maturity status was estimated using predictive equations [17], which may not be as precise as direct physiological assessments. Second, A limitation of the present study is the relatively small sample size and the recruitment of participants from a single Tunisian youth soccer academy. Therefore, the findings may not be fully generalizable to players from other regions or competitive environments, where differences in training exposure, lifestyle, and soccer proficiency could influence both asymmetry patterns and training responses. Future studies including larger, more diverse samples from multiple academies and countries are warranted to confirm the present results. Hence, although the sample size was powered appropriately, larger cohorts would strengthen generalizability and reduce the influence of outliers. Third, while field-based asymmetry assessments are practical, they do not capture the underlying biomechanical or neuromuscular mechanisms of imbalance. Future studies should include kinematic and electromyographic analyses to better understand these adaptations. Additionally, including female athletes and players from different performance tiers would provide a more inclusive view of the effects of plyometric training across populations. In addition, while objective measures such as jump height or force output were not used in-session to quantify intensity, the structured progression and careful selection of jump types served as practical and evidence-based proxies for intensity control in the field-based setting of our intervention. Future research may benefit from incorporating wearable technology or force platforms to further quantify internal and external training load.

Finally, In the present study, post-PHV players demonstrated greater functional improvements following the plyometric training intervention compared with their pre-PHV counterparts, who exhibited limited gains and, in some cases, increased asymmetry. While these findings are consistent with evidence indicating that neuromuscular responsiveness to high-velocity SSC stimuli improves with maturation [7,23,25], they should be interpreted cautiously. The current design did not quantify internal or external training load (e.g., session RPE, jump volume, or time-motion data), which limits the ability to attribute observed changes exclusively to the plyometric intervention. Moreover, developmental factors such as ongoing growth-related neural reorganization and motor coordination variability in pre-PHV athletes [30,31] may have contributed to the divergent adaptations observed between maturity groups. It is also possible that technical learning effects from regular soccer practice influenced movement efficiency and interlimb coordination, thereby affecting asymmetry indices independent of the plyometric stimulus.

Accordingly, while the intervention appears to have enhanced power- and speed-related measures primarily in more mature players, the mechanisms underlying these changes likely reflect an interaction between biological maturation, training exposure, and motor learning processes rather than a direct training effect alone. Future studies should incorporate comprehensive load monitoring and longitudinal follow-up to better isolate the relative contribution of plyometric training from natural developmental trajectories.

## Conclusion

This study concludes that plyometrics training effectively enhances key physical performance aspects—including jumps, change of direction speed, and dynamic balance—in male youth soccer players, with performance gains being more pronounced in post-PHV youth soccer players. This supports existing literature on the benefits of plyometrics and the superior adaptive capacity of more mature individuals. A notable finding, however, was that while overall lower-limb asymmetry did not significantly change, balance-specific asymmetry surprisingly increased within the post-PHV groups. This increase is likely due to a differential enhancement of balance capabilities between the left and right limbs, reflecting a nuanced, perhaps transient, phase of neuromuscular reorganization as more mature athletes integrate new levels of power and strength. Future research should delve deeper into the mechanisms underlying this specific increase in balance asymmetry, particularly investigating long-term effects and optimal training protocols that can mitigate such imbalances while maximizing overall performance improvements across different maturational stages. Furthermore, extending this research to include female athletes, considering sport-specific or positional requirements, and directly assessing the link between plyometrics and injury prevention would significantly enrich the current body of knowledge.

## Supporting information

S1 Data Raw Data. (XLSX)
